# Aqua­[2-(2-pyridylmethyl­imino­meth­yl)phenolato]nickel(II) nitrate monohydrate

**DOI:** 10.1107/S1600536809037027

**Published:** 2009-10-10

**Authors:** Ning Sheng

**Affiliations:** a Department of Chemistry & Chemical Engineering, Jining University, Qufu 273155, People’s Republic of China

## Abstract

In the title compound, [Ni(C_13_H_11_N_2_O)(H_2_O)]NO_3_·H_2_O, the Ni(II) ion is coordinated by one O atom and two N atoms of the Schiff base ligand and the O atom from a water mol­ecule, forming a slightly distorted square-planar geometry. A one-dimensional double-chain structure is formed along [001] by O⋯H—O hydrogen bonds and the Ni⋯O [2.617 (3) Å] inter­actions.

## Related literature

For background to Schiff bases in coordination chemistry, see: Boskovic *et al.* (2003 ▶); Koizumi *et al.* (2005 ▶); Oshiob *et al.* (2005 ▶). For Ni—O and Ni—N bond distances in related structures, see: Wang *et al.* (2007 ▶).
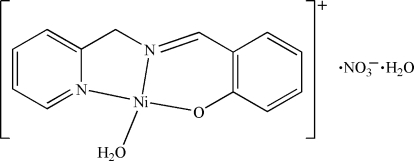


## Experimental

### 

#### Crystal data

[Ni(C_13_H_11_N_2_O)(H_2_O)]NO_3_·H_2_O
*M* *_r_* = 367.99
Triclinic, 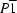

*a* = 7.7885 (13) Å
*b* = 9.0155 (15) Å
*c* = 11.3285 (19) Å
α = 71.244 (2)°
β = 85.846 (3)°
γ = 86.967 (3)°
*V* = 750.9 (2) Å^3^
*Z* = 2
Mo *K*α radiation
μ = 1.33 mm^−1^
*T* = 293 K
0.27 × 0.21 × 0.15 mm

#### Data collection

Bruker APEXII CCD area-detector diffractometer
Absorption correction: multi-scan (*SADABS*; Sheldrick, 2003 ▶) *T*_min_ = 0.716, *T*_max_ = 0.826
3706 measured reflections
2610 independent reflections
2179 reflections with *I* > 2σ(*I*)
*R*_int_ = 0.015

#### Refinement

*R*[*F* ^2^ > 2σ(*F* ^2^)] = 0.033
*wR*(*F* ^2^) = 0.086
*S* = 1.04
2610 reflections
208 parameters
H-atom parameters constrained
Δρ_max_ = 0.36 e Å^−3^
Δρ_min_ = −0.26 e Å^−3^

### 

Data collection: *APEX2* (Bruker, 2004 ▶); cell refinement: *SAINT-Plus* (Bruker, 2001 ▶); data reduction: *SAINT-Plus*; program(s) used to solve structure: *SHELXS97* (Sheldrick, 2008 ▶); program(s) used to refine structure: *SHELXL97* (Sheldrick, 2008 ▶); molecular graphics: *XP* (Sheldrick, 1998 ▶); software used to prepare material for publication: *XP*.

## Supplementary Material

Crystal structure: contains datablocks global, I. DOI: 10.1107/S1600536809037027/hg2552sup1.cif

Structure factors: contains datablocks I. DOI: 10.1107/S1600536809037027/hg2552Isup2.hkl

Additional supplementary materials:  crystallographic information; 3D view; checkCIF report

## Figures and Tables

**Table 1:** Hydrogen-bond geometry (Å, °)

*D*—H⋯*A*	*D*—H	H⋯*A*	*D*⋯*A*	*D*—H⋯*A*
O6—H6*A*⋯O1^i^	0.83	2.12	2.930 (3)	165
O6—H6*B*⋯O3^ii^	0.82	2.00	2.819 (3)	172
O2—H2*B*⋯O5	0.83	2.57	3.009 (3)	114
O2—H2*B*⋯N3	0.83	2.53	3.234 (4)	143
O2—H2*B*⋯O4	0.83	1.85	2.677 (3)	170
O2—H2*A*⋯O6	0.83	1.86	2.681 (3)	168

Symmetry codes: (i) 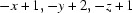; (ii) 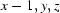.

**Fig. 1.**
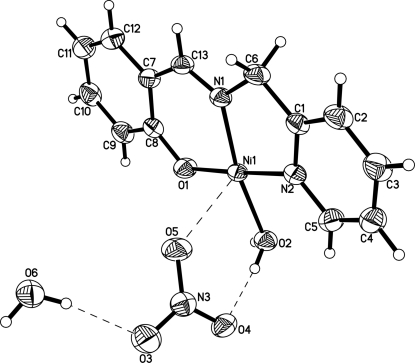
View of the title compound containing the hydrogen bonds with the atom-labelling scheme displacement ellipsoids are drawn at the 30% probability level.

## supplementary crystallographic information

### Comment

Recently, Schiff base ligands, especially the relative flexible unsymmetrical tridentate Schiff base ligands and their hydrogenerated derivatives have been employed to assembly alkoxo-or phenoxo-bridged clusters and polymers with beautiful molecular structures and interesting magnetic properties in the field of coordination chemistry. (Koizumi *et al.*, 2005; Boskovic *et al.*, 2003; Oshiob *et al.*, 2005). Herein, we report the structure of a new nickel complex based on an unsymmetric tridentate Schiff base ligand. The title compound, which is comprised by [Ni(*L*)(H_2_O)]^+^ (*L*=2-(pyridin-2-ylmethyliminomethyl)phenol, nitrate anion and a free water molecule, crystallizes in triclinic cell setting and P-1 space group. The coordination sphere of the Ni ion can be described as slightly distorted square planar, in which three positions are occupied by two N atoms and one O atom from the asymmetric tridentate Schiff base ligand, and the other one coming from the O atom of the solvent water molecule. The bond distances of Ni—O and Ni—N are in the normal range compared to the reported complexes containing the N—Ni—O atoms (Wang *et al.*, 2007). The mean deviation of the plane formed by NiN_2_O_2_ unit is 0.0799 Å, and the Ni ion is only out of the plane 0.0514 Å. The distance between Ni and O5 is only 2.617 Å, indicative of significant interaction between these two atoms. Under the help of these interactions and the O···H—O hydrogen bonds between the O atoms of the water molecules (Table 1), the nitrate ion, and the Schiff base ligand, the asymmetric unit can be linked into one dimensional double chain supermolecular structure.

### Experimental

The Schiff base was synthesized by condensation 2-(aminomethyl)pyridine and 2-hydroxy-benzaldehyde with the ratio 1:1 in methanol. The synthesis of the title complex was carried out by treating Ni(NO_3_)_2_.6H_2_O (1 mmol, 290 mg) and the Schiff-base ligand (1 mmol, 212 mg) in methanol under the stirring condition at room temperature. The filtered solution was left to slowly evaporate in air to obtain single-crystal suitable for X-ray diffraction with a yield of about 202 mg, 55%.

### Refinement

All the H atoms bonded to the C atoms were placed using the HFIX commands in *SHELXL-97*, with C—H distances of 0.93 and 0.96 Å, and were allowed for as riding atoms with *U*_iso_(H) = 1.2*U*_eq_(C). For the H atom of the water molecule, they were found from difference Fourier maps with the O—H bond length restrained to 0.82 Å and was allowed for as riding atoms with *U*_iso_(H) = 1.2*U*_eq_(O).

### Crystal data

**Table:**

[Ni(C_13_H_11_N_2_O)(H_2_O)]NO_3_·H_2_O	*Z* = 2
*M**_r_* = 367.99	*F*(000) = 380
Triclinic, *P*1	*D*_x_ = 1.628 Mg m^−^^3^
Hall symbol: -P 1	Mo *K*α radiation, λ = 0.71073 Å
*a* = 7.7885 (13) Å	Cell parameters from 1525 reflections
*b* = 9.0155 (15) Å	θ = 2.5–25.8°
*c* = 11.3285 (19) Å	µ = 1.33 mm^−^^1^
α = 71.244 (2)°	*T* = 293 K
β = 85.846 (3)°	Block, red-brown
γ = 86.967 (3)°	0.27 × 0.21 × 0.15 mm
*V* = 750.9 (2) Å^3^	

### Data collection

**Table:**

Bruker APEXII CCD area-detector diffractometer	2610 independent reflections
Radiation source: fine-focus sealed tube	2179 reflections with *I* > 2σ(*I*)
graphite	*R*_int_ = 0.015
φ and ω scans	θ_max_ = 25.0°, θ_min_ = 2.4°
Absorption correction: multi-scan (*SADABS*; Sheldrick, 2003)	*h* = −9→9
*T*_min_ = 0.716, *T*_max_ = 0.826	*k* = −7→10
3706 measured reflections	*l* = −13→13

### Refinement

**Table:**

Refinement on *F*^2^	Primary atom site location: structure-invariant direct methods
Least-squares matrix: full	Secondary atom site location: difference Fourier map
*R*[*F*^2^ > 2σ(*F*^2^)] = 0.033	Hydrogen site location: inferred from neighbouring sites
*wR*(*F*^2^) = 0.086	H-atom parameters constrained
*S* = 1.04	*w* = 1/[σ^2^(*F*_o_^2^) + (0.0417*P*)^2^ + 0.2147*P*] where *P* = (*F*_o_^2^ + 2*F*_c_^2^)/3
2610 reflections	(Δ/σ)_max_ = 0.001
208 parameters	Δρ_max_ = 0.36 e Å^−^^3^
0 restraints	Δρ_min_ = −0.26 e Å^−^^3^

### Special details

**Table:**

Geometry. All e.s.d.'s (except the e.s.d. in the dihedral angle between two l.s. planes) are estimated using the full covariance matrix. The cell e.s.d.'s are taken into account individually in the estimation of e.s.d.'s in distances, angles and torsion angles; correlations between e.s.d.'s in cell parameters are only used when they are defined by crystal symmetry. An approximate (isotropic) treatment of cell e.s.d.'s is used for estimating e.s.d.'s involving l.s. planes.
Refinement. Refinement of *F*^2^ against ALL reflections. The weighted *R*-factor *wR* and goodness of fit *S* are based on *F*^2^, conventional *R*-factors *R* are based on *F*, with *F* set to zero for negative *F*^2^. The threshold expression of *F*^2^ > σ(*F*^2^) is used only for calculating *R*-factors(gt) *etc*. and is not relevant to the choice of reflections for refinement. *R*-factors based on *F*^2^ are statistically about twice as large as those based on *F*, and *R*- factors based on ALL data will be even larger.

### Fractional atomic coordinates and isotropic or equivalent isotropic displacement parameters (Å^2^)

**Table:**

	*x*	*y*	*z*	*U*_iso_*/*U*_eq_	
Ni1	0.65546 (5)	0.59075 (4)	0.61677 (3)	0.03862 (14)	
O1	0.6180 (3)	0.7356 (2)	0.45717 (19)	0.0534 (5)	
O2	0.5125 (3)	0.7279 (2)	0.69276 (19)	0.0502 (5)	
H2A	0.4512	0.7909	0.6414	0.060*	
H2B	0.5764	0.7774	0.7215	0.060*	
O3	1.0146 (3)	0.9237 (3)	0.7257 (3)	0.0827 (8)	
O4	0.7495 (3)	0.8666 (3)	0.7775 (2)	0.0646 (6)	
O5	0.8974 (3)	0.7562 (3)	0.6573 (3)	0.0743 (7)	
O6	0.2993 (3)	0.9471 (2)	0.5530 (2)	0.0651 (6)	
H6B	0.2135	0.9322	0.6016	0.078*	
H6A	0.3320	1.0375	0.5370	0.078*	
N1	0.7643 (3)	0.4328 (3)	0.5523 (2)	0.0427 (6)	
N2	0.7069 (3)	0.4333 (3)	0.7799 (2)	0.0455 (6)	
N3	0.8871 (4)	0.8476 (3)	0.7206 (2)	0.0550 (7)	
C1	0.7858 (4)	0.2991 (3)	0.7729 (3)	0.0437 (7)	
C2	0.8364 (4)	0.1813 (4)	0.8778 (3)	0.0586 (9)	
H2	0.8902	0.0897	0.8707	0.070*	
C3	0.8059 (5)	0.2014 (4)	0.9927 (3)	0.0642 (9)	
H3	0.8397	0.1239	1.0646	0.077*	
C4	0.7247 (5)	0.3378 (4)	1.0002 (3)	0.0626 (9)	
H4	0.7027	0.3534	1.0771	0.075*	
C5	0.6771 (4)	0.4494 (4)	0.8938 (3)	0.0583 (8)	
H5	0.6215	0.5407	0.8999	0.070*	
C6	0.8153 (4)	0.2851 (3)	0.6448 (3)	0.0511 (8)	
H6C	0.9361	0.2608	0.6296	0.061*	
H6D	0.7483	0.2008	0.6381	0.061*	
C7	0.7468 (4)	0.5799 (3)	0.3335 (3)	0.0461 (7)	
C8	0.6592 (4)	0.7162 (3)	0.3475 (3)	0.0452 (7)	
C9	0.6142 (4)	0.8354 (4)	0.2387 (3)	0.0588 (9)	
H9	0.5550	0.9250	0.2453	0.071*	
C10	0.6560 (5)	0.8222 (4)	0.1225 (3)	0.0629 (9)	
H10	0.6235	0.9025	0.0519	0.075*	
C11	0.7465 (5)	0.6902 (4)	0.1084 (3)	0.0643 (9)	
H11	0.7764	0.6833	0.0293	0.077*	
C12	0.7902 (4)	0.5718 (4)	0.2126 (3)	0.0575 (8)	
H12	0.8500	0.4836	0.2037	0.069*	
C13	0.7934 (4)	0.4472 (3)	0.4359 (3)	0.0457 (7)	
H13	0.8501	0.3636	0.4169	0.055*	

### Atomic displacement parameters (Å^2^)

**Table:**

	*U*^11^	*U*^22^	*U*^33^	*U*^12^	*U*^13^	*U*^23^
Ni1	0.0453 (2)	0.0280 (2)	0.0426 (2)	0.00371 (14)	0.00055 (15)	−0.01290 (15)
O1	0.0696 (14)	0.0374 (11)	0.0513 (13)	0.0101 (10)	−0.0023 (11)	−0.0135 (10)
O2	0.0555 (12)	0.0402 (11)	0.0564 (13)	0.0045 (9)	−0.0031 (10)	−0.0186 (10)
O3	0.0580 (16)	0.108 (2)	0.097 (2)	−0.0215 (15)	−0.0002 (14)	−0.0517 (18)
O4	0.0579 (14)	0.0783 (17)	0.0680 (15)	−0.0067 (12)	0.0113 (12)	−0.0402 (14)
O5	0.0684 (16)	0.0642 (15)	0.104 (2)	0.0061 (12)	0.0087 (14)	−0.0506 (16)
O6	0.0666 (15)	0.0451 (13)	0.0814 (17)	0.0045 (11)	0.0029 (13)	−0.0196 (12)
N1	0.0493 (14)	0.0332 (12)	0.0459 (14)	0.0027 (10)	−0.0025 (11)	−0.0138 (11)
N2	0.0533 (15)	0.0344 (13)	0.0489 (15)	−0.0009 (11)	−0.0015 (11)	−0.0140 (11)
N3	0.0629 (18)	0.0470 (16)	0.0534 (17)	0.0057 (13)	−0.0081 (14)	−0.0137 (13)
C1	0.0502 (17)	0.0327 (15)	0.0477 (17)	−0.0004 (12)	−0.0062 (14)	−0.0115 (13)
C2	0.077 (2)	0.0391 (17)	0.059 (2)	0.0045 (16)	−0.0157 (17)	−0.0128 (16)
C3	0.089 (3)	0.0478 (19)	0.052 (2)	−0.0025 (18)	−0.0216 (18)	−0.0066 (16)
C4	0.086 (3)	0.056 (2)	0.0450 (19)	−0.0036 (18)	−0.0088 (17)	−0.0147 (17)
C5	0.075 (2)	0.0503 (19)	0.051 (2)	0.0010 (16)	0.0011 (17)	−0.0200 (16)
C6	0.065 (2)	0.0321 (15)	0.0563 (19)	0.0097 (14)	−0.0091 (16)	−0.0153 (14)
C7	0.0525 (18)	0.0422 (17)	0.0462 (17)	−0.0014 (14)	−0.0051 (14)	−0.0174 (14)
C8	0.0491 (17)	0.0373 (16)	0.0490 (18)	−0.0039 (13)	−0.0041 (14)	−0.0126 (14)
C9	0.071 (2)	0.0417 (18)	0.060 (2)	−0.0040 (16)	−0.0066 (17)	−0.0100 (16)
C10	0.082 (2)	0.054 (2)	0.046 (2)	−0.0157 (18)	−0.0108 (17)	−0.0043 (16)
C11	0.082 (3)	0.067 (2)	0.047 (2)	−0.0143 (19)	−0.0020 (17)	−0.0204 (18)
C12	0.064 (2)	0.060 (2)	0.054 (2)	−0.0020 (16)	−0.0017 (16)	−0.0248 (17)
C13	0.0476 (17)	0.0392 (16)	0.0542 (19)	0.0031 (13)	−0.0008 (14)	−0.0215 (14)

### Geometric parameters (Å, °)

**Table:**

Ni1—O1	1.891 (2)	C3—C4	1.377 (5)
Ni1—N1	1.931 (2)	C3—H3	0.9300
Ni1—O2	1.9777 (19)	C4—C5	1.359 (4)
Ni1—N2	1.987 (2)	C4—H4	0.9300
O1—C8	1.324 (3)	C5—H5	0.9300
O2—H2A	0.8290	C6—H6C	0.9700
O2—H2B	0.8324	C6—H6D	0.9700
O3—N3	1.251 (3)	C7—C12	1.411 (4)
O4—N3	1.243 (3)	C7—C8	1.422 (4)
O5—N3	1.251 (3)	C7—C13	1.425 (4)
O6—H6B	0.8225	C8—C9	1.402 (4)
O6—H6A	0.8261	C9—C10	1.374 (5)
N1—C13	1.287 (4)	C9—H9	0.9300
N1—C6	1.462 (4)	C10—C11	1.399 (5)
N2—C5	1.347 (4)	C10—H10	0.9300
N2—C1	1.350 (4)	C11—C12	1.363 (5)
C1—C2	1.381 (4)	C11—H11	0.9300
C1—C6	1.497 (4)	C12—H12	0.9300
C2—C3	1.374 (5)	C13—H13	0.9300
C2—H2	0.9300		
			
O1—Ni1—N1	94.39 (9)	C3—C4—H4	120.3
O1—Ni1—O2	89.12 (8)	N2—C5—C4	122.8 (3)
N1—Ni1—O2	170.48 (9)	N2—C5—H5	118.6
O1—Ni1—N2	176.56 (9)	C4—C5—H5	118.6
N1—Ni1—N2	82.56 (10)	N1—C6—C1	109.4 (2)
O2—Ni1—N2	94.14 (9)	N1—C6—H6C	109.8
C8—O1—Ni1	127.20 (18)	C1—C6—H6C	109.8
Ni1—O2—H2A	111.9	N1—C6—H6D	109.8
Ni1—O2—H2B	109.2	C1—C6—H6D	109.8
H2A—O2—H2B	109.2	H6C—C6—H6D	108.2
H6B—O6—H6A	110.5	C12—C7—C8	119.4 (3)
C13—N1—C6	118.2 (2)	C12—C7—C13	116.9 (3)
C13—N1—Ni1	125.4 (2)	C8—C7—C13	123.6 (3)
C6—N1—Ni1	116.37 (18)	O1—C8—C9	118.8 (3)
C5—N2—C1	117.8 (3)	O1—C8—C7	123.5 (3)
C5—N2—Ni1	127.0 (2)	C9—C8—C7	117.7 (3)
C1—N2—Ni1	115.2 (2)	C10—C9—C8	121.2 (3)
O4—N3—O5	120.7 (3)	C10—C9—H9	119.4
O4—N3—O3	118.9 (3)	C8—C9—H9	119.4
O5—N3—O3	120.4 (3)	C9—C10—C11	121.2 (3)
N2—C1—C2	122.1 (3)	C9—C10—H10	119.4
N2—C1—C6	116.1 (2)	C11—C10—H10	119.4
C2—C1—C6	121.8 (3)	C12—C11—C10	118.8 (3)
C3—C2—C1	119.0 (3)	C12—C11—H11	120.6
C3—C2—H2	120.5	C10—C11—H11	120.6
C1—C2—H2	120.5	C11—C12—C7	121.6 (3)
C2—C3—C4	119.1 (3)	C11—C12—H12	119.2
C2—C3—H3	120.5	C7—C12—H12	119.2
C4—C3—H3	120.5	N1—C13—C7	125.9 (3)
C5—C4—C3	119.3 (3)	N1—C13—H13	117.1
C5—C4—H4	120.3	C7—C13—H13	117.1

### Hydrogen-bond geometry (Å, °)

**Table:**

*D*—H···*A*	*D*—H	H···*A*	*D*···*A*	*D*—H···*A*
O6—H6A···O1^i^	0.83	2.12	2.930 (3)	165
O6—H6B···O3^ii^	0.82	2.00	2.819 (3)	172
O2—H2B···O5	0.83	2.57	3.009 (3)	114
O2—H2B···N3	0.83	2.53	3.234 (4)	143
O2—H2B···O4	0.83	1.85	2.677 (3)	170
O2—H2A···O6	0.83	1.86	2.681 (3)	168

Symmetry codes: (i) −*x*+1, −*y*+2, −*z*+1; (ii) *x*−1, *y*, *z*.
